# Failure to deactivate the default mode network indicates a possible endophenotype of autism

**DOI:** 10.1186/2040-2392-3-15

**Published:** 2012-12-03

**Authors:** Michael D Spencer, Lindsay R Chura, Rosemary J Holt, John Suckling, Andrew J Calder, Edward T Bullmore, Simon Baron-Cohen

**Affiliations:** 1 Department of Psychiatry, Autism Research Centre, University of Cambridge, Douglas House, 18b Trumpington Road, Cambridge, CB2 8AH, UK; 2 Department of Psychiatry, Herchel Smith Building for Brain and Mind Sciences, Cambridge Biomedical Campus, University of Cambridge, Cambridge, CB2 0SZ, UK; 3 MRC Cognition and Brain Sciences Unit, 15 Chaucer Road, Cambridge, CB2 7EF, UK

**Keywords:** Autism, Default mode network, Functional MRI, Endophenotype

## Abstract

**Background:**

Reduced activity during cognitively demanding tasks has been reported in the default mode network in typically developing controls and individuals with autism. However, no study has investigated the default mode network (DMN) in first-degree relatives of those with autism (such as siblings) and it is not known whether atypical activation of the DMN is specific to autism or whether it is also present in unaffected relatives. Here we use functional magnetic resonance imaging to investigate the pattern of task-related deactivation during completion of a visual search task, the Embedded Figures Task, in teenagers with autism, their unaffected siblings and typically developing controls.

**Findings:**

We identified striking reductions in deactivation during the Embedded Figures Task in unaffected siblings compared to controls in brain regions corresponding to the default mode network. Adolescents with autism and their unaffected siblings similarly failed to deactivate regions, including posterior cingulate and bilateral inferior parietal cortex.

**Conclusions:**

This suggests that a failure to deactivate these regions is a functional endophenotype of autism, related to familial risk for the condition shared between individuals with autism and their siblings.

## Findings

### Background

Mounting evidence suggests that in the typical brain there are consistent patterns of increased neural activity during the ‘resting state’, a condition where no active task is being performed by the participant [1], and consistent patterns of reduced activity during cognitively demanding tasks in the default mode network (DMN) [2]. Furthermore, reduced deactivation within DMN structures has been reported in individuals with autism [3].

To our knowledge no studies have investigated resting state activity or task-related deactivation in first-degree relatives of those with autism (such as siblings) and it is not known whether atypical activation of the DMN is specific to autism or whether it is also present in unaffected first-degree family members. Identifying whether atypical DMN activation is a marker of familial risk for autism (and therefore related to shared genetic risk among siblings) would shed new light on the genetic and neural building blocks that underpin autism.

Siblings of individuals with autism have a higher risk of developing the condition, with a prevalence that has been estimated to be as high as twenty times greater than in the general population [4-6]. For an overview of the rationale for endophenotype research in autism, see a recent report from our laboratory [7].

Here we investigate the pattern of task-related deactivation during completion of the Embedded Figures Task (EFT), a task that requires the visual inspection of a complex pattern in order to search for a component shape located within it. We recently reported a functional magnetic resonance imaging (fMRI) investigation of task-related activation during the EFT (that is, activation associated with visual inspection versus a simpler control task) demonstrating an endophenotype of autism in terms of an atypical pattern of activation in teenagers with autism and their unaffected siblings, but not present in controls with no family history of autism [8]. Data already collected during the above task present an opportunity for the investigation of task-related deactivation in autism by analyzing the data to determine the pattern of reduced brain activity associated with the complex visual inspection versus the simpler control task.

## Methods

### Participants

We studied 40 adolescents (12 to 18 years old) with an autism spectrum condition (ASC) diagnosed with either autism or Asperger syndrome (AS), 40 unaffected siblings and 40 typically developing controls. All ASC participants met Diagnostic and Statistical Manual of Mental Disorders, 4th edition (DSM-IV) criteria [9] for autism or AS and were positive on both the Autism Diagnostic Interview – Revised (ADI-R) [10] and the Autism Diagnostic Observation Schedule – Generic (ADOS-G) [11].

Recruitment of this cohort was as previously described [7]. All siblings and controls scored below threshold on a screening tool for ASC, the Social Communication Questionnaire (SCQ) [12]. All siblings were full biological siblings of the ASC participants (based on parental report); controls had no history of an ASC and no first- or second-degree relative with an ASC. Exclusion criteria were full-scale intelligence quotient (IQ) less than 70 as measured using the Wechsler Abbreviated Scale of Intelligence (WASI) [13], any psychiatric diagnosis (other than an ASC in the autism group) or any history of psychotropic medication, seizures, head injury, intracranial surgery or drug abuse. The study was given ethical approval by the Cambridgeshire 1 Research Ethics Committee, and all participants and their parents provided written informed consent.

### Demographic characteristics of participant groups

Demographic characteristics of the overall sample (n = 120) [7] and the subset that completed the EFT (n = 118) [8] have been previously described. The 118 participants included in this study comprised an ASC group (34 males, 4 females; mean age 14.61 years (SD: 1.74); mean IQ 107.11 (range: 81 to 146; SD: 16.0)), a sibling group (12 males, 28 females; mean age 14.83 years (SD: 2.14); mean IQ 113.1 (range: 88 to 133; SD: 10.1)) and a control group (20 males, 20 females; mean age 15.06 years (SD: 1.63); mean IQ 112.4 (range: 83 to 136; SD: 11.1). The ASC group had mean ADOS-G total score (the sum of subdomains A and B) of 12.0 (SD: 4.25), and mean ADI-R total score (the sum of subdomains A, B and C) of 45.7 (SD: 9.48). Groups did not differ in terms of mean age (ANOVA, *F*(2,115) = 0.553, *P* = 0.577) or IQ (ANOVA, *F*(2,115) = 2.622, *P* = 0.077). Furthermore, while males were over-represented in the ASC group, siblings and controls did not differ in terms of sex (χ^2^ = 3.333, df = 1, *P* = 0.110).

### Embedded figures task protocol

The EFT is a visual search task previously described in fMRI studies of adolescents with ASC [14] and typical volunteers [15]. The version of the task employed here [8] comprised two conditions, an EFT condition and a control task (CT) condition. The EFT condition presented the participant with a stimulus comprising a small target figure and a larger pattern, and the participant was asked to decide whether or not the target figure was present inside the larger pattern. The CT condition presented a stimulus similarly comprising a small target figure and a larger pattern; however, part of the larger pattern was highlighted and the participant was asked to decide whether or not the target figure was the same as the highlighted part of the larger pattern. Examples of stimuli and details of stimulus presentation have been previously described [8]. Performance data in terms of mean accuracy and reaction time on the EFT and CT, and the relative difference in accuracy or reaction time between the two conditions have already been reported [8], demonstrating that groups did not differ in mean accuracy or reaction time on either the EFT or CT, and did not differ in terms of the relative difference in accuracy or reaction time between the two conditions. Although a number of non-imaging studies [16,17] have replicated Shah and Frith’s [18] original report of superior EFT performance in autism, the demonstrated lack of a performance difference on this task is concordant with findings of previous fMRI studies investigating the EFT which reported that individuals with autism perform similarly to controls [14,19,20].

### Imaging protocol

All scans were acquired on a Siemens Tim Trio 3-T system (Siemens Healthcare, Erlangen, Germany). Functional images were acquired with a gradient echo planar imaging sequence (repetition time (TR) = 2,000 ms; echo time (TE) = 30 ms; flip angle = 78°; voxel size = 3 × 3 × 3 mm; field of view = 192 × 192 mm; 64 × 64 acquisition matrix). In all, 32 slices were acquired descending in the transverse plane (slice thickness = 3 mm, slice gap = 25%). Each volume was acquired over two seconds and the first three volumes were discarded to avoid equilibration effects.

### Imaging data analysis

Preprocessing and first-level analyses were performed in SPM8 (Wellcome Department of Cognitive Neurology, London, UK) implemented as previously described [7,8], a standard pipeline comprising sinc interpolation to correct for the acquisition of different brain slices at different times, coregistration of Echo Planar Imaging (EPI) and structural scans, normalization to Montreal Neurological Institute (MNI) space [21] and smoothing using a Gaussian kernel of 10 mm FWHM (full width at half maximum). For each subject fMRI responses were modelled using a canonical hemodynamic response function and the general linear model was employed to perform a first level, within-participants analysis on the functional data from each subject individually for the primary contrast (CT minus EFT), with spatial realignment parameters entered as covariates.

**Figure 1:**
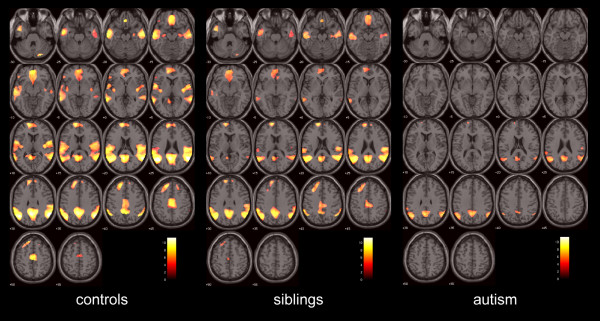
**Task-related deactivations - the neural response to the control task versus the Embedded Figures Task condition, in controls (n = 40), unaffected siblings (n = 40) and adolescents with autism (n = 38).** Activation maps indicate neural response to CT versus EFT conditions, corrected for multiple comparisons at whole brain *P* <0.05 FWE-corrected overlaid onto the canonical MNI152 template brain image (axial section, *z*-coordinate indicated in MNI space), with the colored bar indicating the T-value of the plotted activation differences. CT, control task; EFT, Embedded Figures Task; FWE, family-wise error; MNI, Montreal Neurological Institute.

To characterize the patterns of activation within the brain in the three participant groups, the first-level contrast images for each study group were taken through to a second-level analysis employing a random-effects model, with age and sex specified as covariates. Group-level activation maps (Figure 1) were generated with a global threshold set at *P* <0.05 following correction for multiple comparisons on a whole brain level family-wise error (FWE) basis, and with a cluster extent (*k*_*E*_) threshold set at 10 voxels.

We employed a conjunction analysis to determine commonalities in significant deactivation across all three groups, employing whole brain level FWE-correction for multiple comparisons and a *k*_*E*_ threshold of 10 voxels. In order to investigate the hypothesis that DMN deactivation is an endophenotype for autism and, thus, that siblings would be impaired to an intermediate degree (relative to those with autism and controls) we defined regions of interest (ROI) as the clusters of FWE-corrected *P* <0.05 significant activation common to all three groups as identified by the conjunction analysis and employed MarsBar [22] to extract mean activations for the primary contrast (CT minus EFT) for each subject for each ROI. We used PASW Statistics 18, Release Version 18.0.0 (SPSS, Inc., Chicago, IL, USA), to conduct analyses of variance to measure the overall effect of group on the deactivation data (CT minus EFT) for each ROI. Age and sex were modelled as covariates in all analyses. Similarly we employed analyses of variance to investigate autism versus control, control versus sibling and autism versus sibling differences, again taking age and sex as covariates. We investigated linear trend effects across the three groups using polynomial regression and, where a statistically significant linear effect was found, we examined the quadratic effect to confirm that this was non-significant. We plotted the mean activation contrast estimate (expressed in arbitrary units ± standard error of the mean) for the three study groups.

## Results

We determined regions that were significantly deactivated during the EFT condition. These were measured as regions that were more strongly activated during the CT condition than the EFT condition (CT>EFT), separately in all three groups after correcting for multiple comparisons using FWE correction on a whole brain level (Figure 1 and Additional file 1: Table S1). In controls, strong deactivations (that is, greater activation to CT versus EFT) were found in a network of brain areas dominated by regions corresponding to the DMN. As indicated in Figure 1, the patterns of deactivation in the sibling and autism groups were considerably less extensive than in the control group, the reduction in deactivation being particularly striking in the frontal regions. Clearly evident also on visual inspection of Figure 1 is the trend whereby deactivation appears somewhat reduced in the sibling group, which appears to show an intermediate degree of deactivation to that seen in the other two groups, with the impairment in deactivation appearing particularly marked in the autism group.

**Figure 2:**
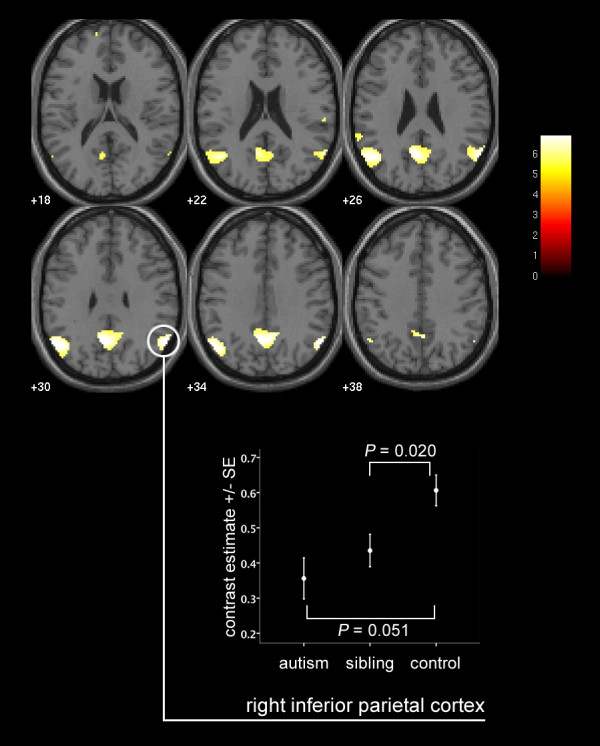
**Commonalities in task-related deactivation across the three groups.** Activation map indicates results of conjunction analysis, signifying commonalities in significant neural response to CT versus EFT conditions across the three groups, corrected for multiple comparisons at whole brain *P* <0.05 FWE-corrected overlaid onto the canonical MNI152 template brain image (axial section, *z*-coordinate indicated in MNI space), with the colored bar indicating the T-value of the plotted activation differences. The graph indicates activation differences (means ± s.e.m.) between the fMRI response in the CT and EFT conditions. Note that the *P* values indicated on the graph additionally take into account age and sex as covariates, and hence do not directly correspond to the degree of apparent separation of error bars. CT, control task; EFT, Embedded Figures Task; fMRI, functional magnetic resonance imaging; FWE, family-wise error; MNI, Montreal Neurological Institute; s.e.m., standard error of the mean.

As indicated in Figure 2 and Table 1, conjunction analysis demonstrated commonalities in the pattern of deactivation across the three groups.After FWE-correction for multiple comparisons on a whole brain level, commonalities of significant deactivation were found in the right inferior parietal cortex (RIPC; *P* <0.001), posterior cingulate cortex (PCC; *P* <0.001), left inferior parietal cortex (LIPC; *P* <0.001), left superior temporal sulcus (STS) (*P* = 0.001), left anterior prefrontal cortex (*P* = 0.006) and right STS (*P* = 0.014).

**Table 1:** Conjunction analysis of common deactivations across autism, sibling and control groups for control task versus embedded figures task

**MNI coordinates**			***P*-value (FWE-corrected)**	***Z*-score**	**Cluster size**	**Region**
***x***	***y***	***z***			***k***_***E***_**(voxels)**	
60	−60	28	<0.001	6.29	494	Right inferior parietal cortex
−4	−60	30	<0.001	6.14	978	Posterior cingulate cortex
−46	−64	28	<0.001	5.97	811	Left inferior parietal cortex
−66	−42	28	0.001	5.13	81	Left superior temporal sulcus
−14	64	16	0.006	4.79	40	Left anterior prefrontal cortex
58	−24	22	0.014	4.84	33	Right superior temporal sulcus

Deactivated brain regions, corresponding MNI coordinates, cluster sizes, *Z*-scores and *P*-values. *P*-values are expressed following family wise error correction for multiple comparisons on a whole brain level; results thresholded at *k*_*E*_ ≥10. FWE, family wise error; MNI, Montreal Neurological Institute.

Reduced deactivation compared to controls was found in sibling and autism groups within a number of the regions of task-related deactivation in controls listed in Table 1. Deactivation in unaffected siblings was reduced compared to controls in the RIPC (*P* = 0.020; F = 5.675) and trends towards significant reductions were found in the LIPC (*P* = 0.061; F = 3.625) and PCC (*P* = 0.064; F = 3.528). Trends towards significant reductions in deactivation in participants with autism compared to controls were found in the RIPC (*P* = 0.051; F = 3.952) and PCC (*P* = 0.057; F = 3.755). A significant effect of group was found in the RIPC (*P* = 0.040; F = 3.313) and, as a trend to significance, in the PCC (*P* = 0.070; F = 2.724). Furthermore, the RIPC (*P* = 0.043) and PCC (P = 0.054) demonstrated a polynomial regression linear trend effect with no significant quadratic component (Table 2 and Figure 2). For all areas where significant between-group differences in deactivation were found, the effects of sex and age were non-significant.

**Table 2:** Between-group differences in deactivation (that is, CT>EFT) – all analyses take age and sex as covariates

**Region of commonality of significant deactivation in all three groups**	**Between-group differences**			**Effect of group (across all three groups)**	**Polynomial regression linear trend effect**
	***P*-value (*F*statistic)**				
	**Control versus sibling**	**Control versus autism**	**Sibling versus autism**	***P*-value (*F*statistic)**	***P*-value**
**CT versus EFT**					
RIPC	0.020 (5.675)	0.051 (3.952)	NS	0.040 (3.313)	0.043
PCC	0.064 (3.528)	0.057 (3.755)	NS	0.070 (2.724)	0.054
LIPC	0.061 (3.625)	NS	NS	NS	NS
LSTS	NS	NS	NS	NS	NS
LAPFC	NS	NS	NS	NS	NS
RSTS	NS	NS	NS	NS	NS

CT, control task; EFT, Embedded Figures Task; LAPFC, left anterior prefrontal cortex; LIPC, left inferior parietal cortex; LSTS, left superior temporal sulcus; NS, not significant; PCC, posterior cingulate cortex; RIPC, right inferior parietal cortex; RSTS, right superior temporal sulcus.

Strikingly, within none of these areas did deactivation differ significantly between participants with autism and their unaffected siblings. These results provide evidence that task-related deactivation in these DMN structures demonstrates sibling versus control differences in addition to case versus control differences, and that reduced task-related deactivation in these DMN areas is, therefore, a candidate endophenotype for autism, and a marker for the shared familial risk for autism in both cases and unaffected family members.

The above analysis was repeated with the additional covariate of IQ, and the sibling versus control differences were robust to this additional covariate (in fact, they were marginally more significant), demonstrating reduced deactivation in siblings versus controls in the RIPC (*P* = 0.019; F = 5.800) and trends towards significant reductions in the left IPC (*P* = 0.053; F = 3.865) and PCC (*P* = 0.059; F = 3.674). With IQ as a covariate, the overall effect of group was still significant in the RIPC (*P* = 0.050; F = 3.313), although the autism versus control and polynomial regression linear trend findings were no longer significant.

## Discussion

The key areas of deactivation common to the three groups, LIPC, RIPC and PCC, are concordant with DMN nodes identified in a recent meta-analysis [23], the consensus of 62 separate papers that included data from a total of 840 participants, and a review paper [24]. We identified a striking reduction in the deactivation in these regions in adolescents with autism and their unaffected siblings compared to controls with no family history of autism, during a standard fMRI visual search paradigm, the Embedded Figures Task. The reduction in deactivation was evident in bilateral IPC and PCC, extending through other brain areas including prefrontal cortex, and bilateral middle and superior temporal gyri. This pattern of greatly reduced deactivation relative to controls, strongly evident in participants with autism and evident to an intermediate but nonetheless statistically significant degree in siblings, suggests a possible endophenotype of autism, a neurobiological marker for the familial risk of the condition.

Kennedy and colleagues [3] demonstrated the failure of task-related deactivation in participants with autism during a Stroop task and noted this atypical effect in a network of brain areas including the PCC, anterior cingulate cortex and medial prefrontal cortex. They hypothesized that these findings were due to a lack of activity in these structures during the resting state.By characterizing task-related deactivation in unaffected siblings for the first time, we have been able to show that a marked impairment of deactivation is also evident in siblings, who differ from controls only in terms of bearing the familial genetic risk for autism, and that between-group differences are suggestive of a possible functional endophenotype of autism.

An endophenotype is a heritable feature associated with a condition, present in affected individuals regardless of whether their condition is manifested, which co-segregates with the condition in families and which is present in unaffected family members at a higher rate than in the general population [25]. Endophenotypes occupy a hypothesized intermediate position between genotype and phenotype. As no pathological mechanism has as yet been established, linking deactivation with the autistic phenotype, the interpretation of these findings as evidence of an endophenotype of autism must currently be regarded as preliminary.

A limitation of this work is that, by only investigating ROIs that show activation at the FWE-corrected whole brain level in all three groups, areas that do not achieve this threshold in one or two of the groups are missed by this conservative approach. As Figure 1 suggests, the medial prefrontal cortex may be one such area that may show a graded group difference with reduced activation in the sibling group and with activation not surviving FWE-correction in the ASC group. It may be that failure to deactivate the medial prefrontal cortex is also a potential functional endophenotype of autism. Further investigation of DMN function within this brain area in siblings, perhaps in resting state studies, would, therefore, be of great interest.

We did not select sibling pairs on the basis of sex and, therefore, it is unsurprising that there is an over-representation of males in the ASC group, in keeping with well-established sex ratios in high-functioning autism and AS. Sex is, however, an unlikely explanation for our results, as we have demonstrated a polynomial linear trend across all three study groups (autism<siblings<controls), whereas the sex differences between autism versus siblings, and siblings versus control groups are in opposite directions. Furthermore, our findings are unlikely to be driven by sex because, for every analysis of variance where we detected a significant difference between groups in terms of deactivation, we confirmed that there was no significant effect of sex.

A further potential limitation is that, while we demonstrated that although the key findings of siblings versus control deactivation differences were robust to the inclusion of IQ as a covariate, the autism versus control and polynomial regression linear trend results were no longer significant after covarying for IQ. However, there is a well-established association between autism and intellectual disability, and this may in part account for the failure to identify significant autism versus control differences after covarying for IQ. Of note, this limitation does not apply to the sibling versus control differences that are particularly central to our demonstration of a neurobiological marker associated with the familial risk for autism in unaffected siblings compared to controls with no family history of autism.

Another limitation of the study is that, although efforts were made to recruit the controls from demographically similar areas to the study sample as previously described [7], no further demographic information relating to potential geographic or socio-economic confounds is available.

Future work is indicated to confirm whether the effect reported here is a consequence of a generalized abnormality of the DMN in autism or is specific to the EFT. On the basis of prior evidence using an unrelated task [3] we would suggest that our findings reflect a general, task-unrelated feature of the DMN in autism. However, to further investigate this issue, further work involving other tasks would indicate whether the effects reported here are also seen with other, unrelated paradigms.

It has been suggested that DMN activity at rest is related to cognitive processes such as introspection, theory of mind, self-awareness, mind wandering and awareness of one’s surroundings [26-28]. Since such thoughts could be distracting and detrimental to performance under conditions of high cognitive load, it is plausible that task-related deactivation of the DMN serves to suppress potentially distracting information, thus facilitating attention.

Our findings offer speculative explanations for aspects of the clinical presentation of autism, including self-reported sensory hypersensitivity [9] and comorbid attention-deficit hyperactivity disorder (ADHD) [29]. It is tempting to speculate that these symptoms could be underpinned by the failure to deactivate areas of the DMN and the failure to suppress awareness of surroundings.

## Conclusion

Deactivation of the default mode network is strikingly impaired in adolescents with autism and their unaffected siblings, compared to controls with no family history of autism. We propose that the failure to deactivate this system is an endophenotype of autism, related to familial risk for the condition.

## Abbreviations

ADHD: Attention deficit hyperactivity disorder; ADI-R: Autism Diagnostic Interview – Revised; ADOS-G: Autism Diagnostic Observation Schedule – Generic; ANOVA: Analysis of variance; AS: Asperger syndrome; ASC: Autism spectrum condition; CT: Control task; DMN: Default mode network; DSM-IV: Diagnostic and Statistical Manual of Mental Disorders Fourth Edition; EFT: Embedded Figures Task; FWE: Family-wise error; FWHM: Full width at half maximum; IQ: Intelligence quotient; LIPC: Left inferior parietal cortex; MNI: Montreal Neurological Institute; MRI: Magnetic resonance imaging; PCC: Posterior cingulate cortex; RIPC: Right inferior parietal cortex; ROI: Region of interest; SCQ: Social Communication Questionnaire; SD: Standard deviation; SPM8: Statistical Parametric Mapping 8; STS: Superior temporal sulcus; TE: Echo time; TR: Repetition time; WASI: Wechsler Abbreviated Scale of Intelligence.

## Competing interests

ETB is employed half-time by the University of Cambridge and half-time by GlaxoSmithKline plc. None of the other authors have any other biomedical financial interests or potential conflicts of interest.

## Authors’ contributions

MDS, ETB and SBC designed the study. RJH, LRC and MDS collected the data. MDS analyzed the data and wrote the first draft of the manuscript. All authors contributed to the interpretation of data, revision of the manuscript and approved the final version.

## Supplementary Material

Additional file 1 **Table S1.** Main deactivations to Embedded Figures versus control task. Brain regions activated significantly more strongly to control task versus Embedded Figures Task, corresponding MNI coordinates, cluster sizes, *Z*-scores and *P*-values. All analyses are corrected for multiple comparisons, and *P*-values are expressed following whole brain level family-wise error (FWE) correction at the threshold of *P* <0.05. CT, control task; DMPFC, dorsomedial prefrontal cortex; EFT, Embedded Figures Task; ITG ,inferior temporal gyrus; LIPC, left inferior parietal cortex; MNI, Montreal Neurological Institute; MTG, middle temporal gyrus; PCC, posterior cingulate cortex; RIPC, right inferior parietal cortex; STS, superior temporal sulcus; VACC ,ventral anterior cingulate cortex; VLPFC ,ventrolateral prefrontal cortex;VMPFC, ventromedial prefrontal cortex. Click here for file
