# Supplementary material for: Failure to deactivate the default mode network indicates a possible endophenotype of autism
Source: Mol Autism. 2012 Dec 3;3:15. doi: 10.1186/2040-2392-3-15 (PMC3539860; doi:10.1186/2040-2392-3-15)
Supplement: Additional file 1 — Table S1. Main deactivations to Embedded Figures versus control task. Brain regions activated significantly more strongly to control task versus Embedded Figures Task, corresponding MNI coordinates, cluster sizes, Z-scores and P-values. All analyses are corrected for multiple comparisons, and P-values are expressed following whole brain level family-wise error (FWE) correction at the threshold of P <0.05. CT, control task; DMPFC, dorsomedial prefrontal cortex; EFT, Embedded Figures Task; ITG ,inferior temporal gyrus; LIPC, left inferior parietal cortex; MNI, Montreal Neurological Institute; MTG, middle temporal gyrus; PCC, posterior cingulate cortex; RIPC, right inferior parietal cortex; STS, superior temporal sulcus; VACC ,ventral anterior cingulate cortex; VLPFC ,ventrolateral prefrontal cortex;VMPFC, ventromedial prefrontal cortex. [file 2040-2392-3-15-S1.pdf]

MNI coordinates			P-value (FWE-corrected)	Z-score	Cluster size	Region
x	*y*	*z*			k_E (voxels)	
**CT > EFT**						
Control group:						
58	-58	28	<0.001	>8	6815	RIPC, right STS, right MTG, right hippocampal body
-44	-62	26	<0.001	>8	7450	LIPC, left STS, left MTG
-4	-56	28	<0.001	>8	4705	PCC
30	-82	-32	<0.001	7.13	54	Right cerebellar hemisphere
0	40	-16	<0.001	7.10	4283	VACC, bilateral VMPFC, bilateral DMPFC
-52	30	-8	0.001	5.16	135	Left VLPFC
-24	-16	-18	0.001	5.15	73	Left hippocampal body
Sibling group:						
-44	-62	26	<0.001	>8	2710	LIPC, left ITG, left postcentral gyrus
56	-58	26	<0.001	7.78	2444	RIPC, right postcentral gyrus
-6	-58	28	<0.001	7.33	2544	PCC
-12	60	18	<0.001	6.34	2751	VACC, bilateral VMPFC, bilateral DMPFC
-42	4	-32	<0.001	6.23	957	Left MTG
30	-82	-32	<0.001	6.22	19	Right cerebellar hemisphere
60	-10	-24	<0.001	5.34	500	Right MTG
10	56	30	0.009	4.71	45	Right DMPFC
68	-44	-4	0.035	4.37	11	Right ITG
Autism group:						
60	-60	28	<0.001	6.29	494	RIPC
-4	-60	30	<0.001	6.14	982	PCC
-46	-64	28	<0.001	5.97	814	LIPC
-66	-42	28	0.001	5.13	81	Left STS
-14	64	16	0.006	4.79	40	Left DMPFC
58	-24	22	0.014	4.60	33	Right STS

Supplementary Table 1 Main deactivations to Embedded Figures versus control task. Brain regions activated significantly more strongly to control task versus Embedded Figures Task, corresponding MNI coordinates, cluster sizes, *Z*-scores and *P*-values. All analyses are corrected for multiple comparisons, and *P*-values are expressed following whole brain level family-wise error (FWE) correction at the threshold of $P<0.05$. MNI – Montreal Neuroimaging Institute; CT – control task; EFT – Embedded Figures Task; RIPC – right inferior parietal cortex; STS – superior

temporal sulcus; MTG – middle temporal gyrus; LIPC – left inferior parietal cortex; PCC – posterior cingulate cortex; VACC – ventral anterior cingulate cortex; VMPFC – ventromedial prefrontal cortex; DMPFC – dorsomedial prefrontal cortex; VLPFC – ventrolateral prefrontal cortex; ITG – inferior temporal gyrus.
